# Using a Robust and Sensitive GFP-Based cGMP Sensor for Real-Time Imaging in Intact *Caenorhabditis elegans*

**DOI:** 10.1534/genetics.119.302392

**Published:** 2019-07-22

**Authors:** Sarah Woldemariam, Jatin Nagpal, Tyler Hill, Joy Li, Martin W. Schneider, Raakhee Shankar, Mary Futey, Aruna Varshney, Nebat Ali, Jordan Mitchell, Kristine Andersen, Benjamin Barsi-Rhyne, Alan Tran, Wagner Steuer Costa, Michelle C. Krzyzanowski, Yanxun V. Yu, Chantal Brueggemann, O. Scott Hamilton, Denise M. Ferkey, Miri VanHoven, Piali Sengupta, Alexander Gottschalk, Noelle L’Etoile

**Affiliations:** * Chemistry and Chemical Biology Graduate Program, University of California, San Francisco, California 94158; † Department of Cell and Tissue Biology, University of California, San Francisco, California 94143; ‡ Department of Molecular Membrane Biology and Neurobiology, The Goethe University, 60323 Frankfurt, Germany; § Neuroscience Graduate Program, Brandeis University, Waltham, Massachusetts 02453; ** Department of Biology, Brandeis University, Waltham, Massachusetts 02454; †† Department of Biological Sciences, San Jose State University, California 95192; ‡‡ Department of Biological Sciences, University at Buffalo, The State University of New York, New York 14260; §§ Center for Neuroscience, University of California, Davis, California 95618

**Keywords:** FlincG3, cGMP, visual reporter, sensory neuron, *C. elegans*

## Abstract

cGMP plays a role in sensory signaling and plasticity by regulating ion channels, phosphodiesterases, and kinases. Studies that primarily used genetic and biochemical tools suggest that cGMP is spatiotemporally regulated in multiple sensory modalities. FRET- and GFP-based cGMP sensors were developed to visualize cGMP in primary cell culture and *Caenorhabditis elegans* to corroborate these findings. While a FRET-based sensor has been used in an intact animal to visualize cGMP, the requirement of a multiple emission system limits its ability to be used on its own as well as with other fluorophores. Here, we demonstrate that a *C. elegans* codon-optimized version of the cpEGFP-based cGMP sensor FlincG3 can be used to visualize rapidly changing cGMP levels in living, behaving *C. elegans*. We coexpressed FlincG3 with the blue-light-activated guanylyl cyclases BeCyclOp and bPGC in body wall muscles, and found that the rate of change in FlincG3 fluorescence correlated with the rate of cGMP production by each cyclase. Furthermore, we show that FlincG3 responds to cultivation temperature, NaCl concentration changes, and sodium dodecyl sulfate in the sensory neurons AFD, ASEL/R, and PHB, respectively. Intriguingly, FlincG3 fluorescence in ASEL and ASER decreased in response to a NaCl concentration upstep and downstep, respectively, which is opposite in sign to the coexpressed calcium sensor jRGECO1a and previously published calcium recordings. These results illustrate that FlincG3 can be used to report rapidly changing cGMP levels in an intact animal, and that the reporter can potentially reveal unexpected spatiotemporal landscapes of cGMP in response to stimuli.

THE canonical second messenger molecule cGMP (cyclic guanosine monophosphate) regulates richly diverse functions in an animal’s nervous system. cGMP signaling underlies the outgrowth of axons and the transduction of light, scent, and other environmental cues to electrical signals in the brain (Potter 2011). Because so many neurobiological processes revolve around cGMP, having a robust, easy to use, visual reporter for cGMP with precise temporal and spatial fidelity is critical to complement the primarily pharmacological, biochemical, and genetic approaches used to study this second messenger’s role in these processes. Such a reporter can be used to illuminate how producers (guanylyl cyclases) and degraders (phosphodiesterases) of cGMP shape the landscape of this cyclic nucleotide in neurons.

Since cGMP is used in diverse cell types as a second messenger, its levels need to be regulated in ways that serve the cells’ distinct functions. cGMP production can be regulated directly by stimuli such as ions, peptides, temperature, and gases that interact with the guanylyl cyclases (GCs) that convert GTP to cyclic GMP (Yu *et al.* 1997; Lucas *et al.* 2000; Ortiz *et al.* 2009; Hallem *et al.* 2011; Couto *et al.* 2013; Smith *et al.* 2013; Singhvi *et al.* 2016). Recent evidence suggests that stimuli such as ions, peptides, Gα, and temperature appear to largely regulate transmembrane receptor guanylyl cyclases (rGCs), while membrane permeable gases such as nitric oxide, carbon dioxide, and oxygen have been shown to directly activate soluble guanylyl cyclases (sGCs) (Lucas *et al.* 2000; Hallem *et al.* 2011; Couto *et al.* 2013). Opposing the activity of GCs are phosphodiesterases (PDEs) that hydrolyze cGMP; they can be regulated by cGMP, cAMP, calcium, kinases, and the γ subunit of heteromeric G proteins (Conti and Beavo 2007; Francis *et al.* 2011; Ahmad *et al.* 2015). cGMP effectors, including cyclic nucleotide-gated channels and kinases, can act rapidly by changing the membrane potential of a cell (*e.g.*, the visual system); they can also have slower, more long-lasting effects on gene expression (Lee *et al.* 2010; Juang *et al.* 2013). Thus, the precise subcellular localization of GC and PDE proteins and their temporally regulated activities are likely to produce a complex and dynamic landscape of varying cGMP levels and restricted localized activation of effector proteins.

In neurobiology, the spatial localization of cGMP signal transduction pathways suggest that spatiotemporal regulation of cGMP could play a role in sensory transduction and plasticity, which is likely to be different in distinct neurons (Mukhopadhyay *et al.* 2008; Gross *et al.* 2015). To this end, the transparent nematode *C. elegans* is an ideal model system to visualize cGMP in neurobiological processes. While the use of genetic manipulations in this animal yielded valuable insights into the role of cGMP in sensory transduction, a visual tool that complements this approach has the potential to reveal how this neuromodulator is spatiotemporally regulated in real time in a multitude of sensory modalities. For instance, mutants lacking either the rGCs GCY-8, GCY-18, and/or GCY-23 or the cyclic nucleotide-gated cation channel TAX-2/TAX-4 in the thermosensory neuron AFD showed that cGMP signaling is required for sensing changes in temperature (Komatsu *et al.* 1996; Kimura *et al.* 2004; Inada *et al.* 2006; Ramot *et al.* 2008; Wang *et al.* 2013; Takeishi *et al.* 2016; Goodman and Sengupta 2018). However, while evidence suggests that these rGCs and TAX-2/TAX-4 are localized to the dendrite tip of AFD, it was unknown whether cGMP levels were also spatially regulated in this neuron (Inada *et al.* 2006; Nguyen *et al.* 2014). Additionally, while mutants lacking distinct, functional rGCs revealed that cGMP signaling is required for sensing specific gustatory cues and NaCl concentration cultivation preference in *C. elegans*, it remained unclear whether this second messenger was spatiotemporally regulated as well (Ortiz *et al.* 2009; Kunitomo *et al.* 2013; Smith *et al.* 2013). Furthermore, while genetic evidence suggests that the cGMP-dependent protein kinase (PKG) PKG-1/EGL-4 and GCs regulate *C. elegans*’ sensitivity to quinine through the flow of cGMP from sensory neurons to the nociceptive neuron ASH through gap junctions (Krzyzanowski *et al.* 2013, 2016), a visual tool could greatly enhance our understanding of the process and its dynamics. In another nociceptive neuron, PHB, genetic evidence suggests that *srb-6*, which encodes a G-protein-coupled receptor (GPCR), is required to sense noxious liquids including sodium dodecyl sulfate (SDS) and dodecanoic acid (Tran *et al.* 2017). Calcium recordings of PHB also suggest that avoidance of isoamyl alcohol is at least partially mediated by TAX-2/TAX-4 (Zou *et al.* 2017). Both of these findings in PHB suggest that cGMP flux is required for sensing some nociceptive cues. Pairing genetic tools that demonstrate the importance of cGMP signaling in these distinct sensory modalities with a tool to visualize cGMP fluxes with precise temporal and spatial fidelity could deepen our understanding of these important processes, and offer a more complete picture of the cGMP landscape and its dynamics in cells. Such a tool will provide an essential, complementary approach to the primarily genetic approaches that have been used to examine cGMP dynamics in this animal (O’Halloran *et al.* 2012).

Though a Förster resonance energy transfer (FRET)-based tool has been used to this end, a single-channel fluorophore tool provides additional flexibility as it would allow for more wavelengths to be used, making it more amenable for visualization with other reporters (*e.g.*, calcium sensors and fluorescent markers for organelles). In the olfactory neuron AWC, this FRET-based sensor showed cilia-compartmentalized cGMP dynamics in response to odorants (Shidara *et al.* 2017). Additionally, Couto *et al.* (2013) found that, in the oxygen-sensing neuron PQR, simultaneous imaging of the cGMP sensor with a calcium sensor in response to a 7–21% increase in oxygen revealed that a decrease in cGMP correlated with an increase in calcium. Interestingly, Couto *et al.* (2013) also suggested that compartmentalization of cGMP levels by the action of a PDE might allow cGMP to increase in one compartment while decreasing in another compartment of the cell. While these studies demonstrate that the FRET-based sensor can be used to visualize the cGMP landscape in neurons, the complexity of a multiple emission system raises a barrier to its use. Thus, having a robust, single fluorophore sensor for cGMP will complement the use of calcium sensors in this animal, providing a way to investigate how the spatiotemporal regulation of cGMP influences neural activity *in vivo*. Such a tool would be maximally efficient for probing the interplay between cGMP and calcium dynamics within sensory compartments in this transparent organism.

Here, we show that the *C. elegans* codon-optimized version of the circularly permuted GFP-based cGMP sensor FlincG3 (Bhargava *et al.* 2013) reports cGMP dynamics *in vivo* in *C. elegans*. We characterize the biochemical and biophysical properties of FlincG3 upon cGMP binding *in vivo* by ectopically expressing the blue-light-activated guanylyl cyclases BeCyclOp and bPGC in muscle cells (Gao *et al.* 2015). Using the FlincG3 reporter, we show for the first time that sensory stimulation of thermosensory, gustatory, and nociceptive neurons triggers cGMP changes. We show that FlincG3 fluorescence increases specifically in response to cultivation temperature in the dendrite tip of the thermosensory neuron AFD, mirroring the changes observed using calcium sensors (Kimura *et al.* 2004; Clark *et al.* 2006). We also demonstrate that FlincG3 fluorescence reliably decreases in response to NaCl concentration upsteps and downsteps in the salt-sensing neurons ASEL and ASER, respectively. This decrease in FlincG3 fluorescence is opposite in sign to previously described calcium transients that showed an *increase* in response to NaCl concentration upsteps and downsteps in ASEL and ASER, respectively (Suzuki *et al.* 2008; Ortiz *et al.* 2009; Luo *et al.* 2014). Importantly, we corroborate these findings with the observation that the fluorescence of the coexpressed red calcium sensor jRGECO1a (Dana *et al.* 2016) increases in response to a NaCl concentration upstep and downstep in ASEL and ASER, respectively. Finally, we demonstrate that FlincG3 fluorescence increases in the phasmid PHB neurons in response to a repulsive stimulus. Our results demonstrate that the GFP-based cGMP sensor FlincG3 is a versatile tool for the study of cGMP dynamics in different sensory modalities in intact animals using a single fluorophore.

## Materials and Methods

### Molecular biology

Details on plasmid construction can be found in supplemental materials and methods.

### Transgenic strains

Transgenic *C. elegans* were obtained by microinjection of DNA into the gonads of nematodes by standard procedures (Fire 1986). *ZX1921 (zxEx895[myo-3p*::*CyclOp*::*SL2*::*mCherry*, *myo-3p*::*FlincG3])*: 15 ng/µl *myo-3p*::CyclOp::SL2::mCherry and 15 ng/µl *myo-3p*::*FlincG3* were microinjected into N2 background worms. *ZX1757 (zxEx893[myo-3p*::*mCherry*, *myo-3p*::*FlincG3]):* 5 ng/µl *myo-3p*::mCherry and 20 ng/µl *myo-3p*::*FlincG3* were microinjected into N2 background worms. *ZX1922 (zxEx896[myo-3p*::*bPAC*::*SL2*::*mCherry*, *myo-3p*::*FlincG3]):* 15 ng/µl *myo-3p*::bPAC::SL2::mCherry and 15 ng/µl *myo-3p*::*FlincG3* were microinjected into N2 background worms. *ZX1756 (zxEx892[myo-3p*::*bPGC*::*SL2*::*mCherry*, *myo-3p*::*FlincG3]):* 15 ng/µl *myo-3p*::bPGC::SL2::mCherry and 15 ng/µl *myo-3p*::*FlincG3* were microinjected into N2 background worms. *PY12100 (AFD FlincG3):* 50 ng/µl *gcy-8p*::*FlincG3*, 5 ng/µl gcy-8:MyrTagRFP and 30 ng/µl unc-122::dsRed were microinjected into N2 background worms. *JZ1994* (ASE FlincG3 in N2 injected with 15 ng/µl FlincG3; behavior shown in Supplemental Material, Figure S7, line 1): 15 ng/µl *flp-6p*::*FlincG3*, 30 ng/µl *flp-6p*::*jRCaMP1b*, and 20 ng/µl ofm-1::GFP were microinjected into N2 background worms. *JZ1996* (ASE FlincG3 in N2 injected with 15 ng/µl FlincG3; behavior shown in Figure S7, line 2): 15 ng/µl *flp-6p*::*FlincG3*, 30 ng/µl *flp-6p*::*jRCaMP1b*, and 20 ng/µl ofm-1::GFP were microinjected into N2 background worms. *JZ1997* (ASE FlincG3 in N2 injected with 15 ng/µl FlincG3; behavior shown in Figure 6B): 15 ng/µl *flp-6p*::*FlincG3*, 30 ng/µl *flp-6p*::*jRCaMP1b*, and 20 ng/µl ofm-1::GFP were microinjected into N2 background worms. *JZ2089* (ASE FlincG3 in N2 injected with 7.5 ng/µl FlincG3): 7.5 ng/µl *flp-6p*::*FlincG3*, 60 ng/µl *flp-6p*::*jRGECO1a*, and 20 ng/µl ofm-1::GFP were microinjected into N2 background worms. *JZ2118* (ASE FlincG3 in *gcy-22*): JZ2089 animals were crossed with OH4839 (*gcy-22**(**tm2364**)*) animals to generate transgenic animals homozygous for *gcy-22**(**tm2364**)*. *PHB FlincG3 in N2*: *MKV937* (*iyEx222* (15 ng/µl *nlp-1p*::*FlincG3* and 45 ng/µl *odr-1p*::*RFP* in N2 background worms)). *ZX1738 (zxEx886[myo-3p*::*bPGC*::*YFP*, *myo-3p*::*tax-2*::*GFP*, *myo-3p*::*tax-4*::*GFP])*: 15 ng/µl *myo-3p*::bPGC::YFP, 5 ng/µl *myo-3p*::tax-2::GFP, and 5 ng/µl *myo-3p*::tax-4::GFP were injected into *lite-1**(**ce314**)* background worms.

*ZX1739 (zxEx887[myo-3p*::*bPGC(K265D)*::*YFP*, *myo-3p*::*tax-2*::*GFP*, *pmyo-3*::*tax-4*::*GFP])*: 15 ng/µl *myo-3p*::bPGC(K265D)::YFP, 5 ng/µl *myo-3p*::tax-2::GFP, and 5 ng/µl *myo-3p*::tax-4::GFP were injected into *lite-1**(**ce314**)* background worms.

### Imaging FlincG3 coexpressed with BeCyclOp, bPGC or bPAC

Transgenic strains were kept in the dark on standard nematode growth media (NGM) plates (5.5 cm diameter; 8 ml NGM) with OP50-1 bacteria with or without all-*trans*-retinal (ATR) at 20°. Plates containing ATR were prepared by spreading 200 µl of OP50-1 culture containing 100 mM of ATR (diluted in ethanol). L4 animals were put on ATR plates overnight and young adults were used for imaging the following day.

For cGMP/cAMP imaging, animals were immobilized on 10% M9 agar pads with polystyrene beads (Polysciences). The fluorescence measurements were performed with a 25× oil objective (25× LCI-Plan/0.8 Imm Corr DIC; Zeiss) on the inverted microscope Axio Observer Z.1 equipped with two high-power light emitting diodes (LEDs; 470 and 590 nm wavelength, KSL 70; Rapp Optoelektronik) coupled via a beam splitter and a double band pass excitation filter permitting wavelengths of 479/21 and 585/19 nm (F74-423 479/585 HC Dualband Filter AHF Analysentechnik) to obtain simultaneous dual-wavelength illumination. DIC microscopy using white light filtered with a red optical filter was used to focus on the body wall muscle cells prior to video acquisition. The 470 and 590 nm excitation were switched on simultaneously after the start of video acquisition. For bPGC experiments, yellow light was used to focus the cells, and, thereafter, the blue illumination was turned on. Fluorescence was acquired by an ORCA-Flash 4.0 sCMOS camera (Hamamatsu) through a DualView beam splitter (DV2; Photometrics) with a 540/25 nm emission filter used for FlincG3 green channel and a 647/57 nm emission filter for mCherry red channel. Videos were acquired using µManager (Edelstein *et al.* 2010), and frames were taken at 100 Hz (corresponding to exposure times of 10 msec) and 20 Hz (for bPGC) with 4 × 4 spatial binning. The optical power was 3.3 mW/mm^2^ at 470 nm and 2.6 mW/mm^2^ at 590 nm.

Image analysis was performed in ImageJ (National Institutes of Health). Regions of interest (ROIs) were drawn around single body wall muscle cells that did not show major movement, and a region outside the animals was chosen as background ROI. The mean fluorescence intensity of the ROIs for both channels was analyzed with ImageJ. Background subtracted values were used to calculate the change in fluorescence intensity for each time point: ΔF/F_0_ ((F−F_0_)/F_0_), where F represents the intensity at this time point, and F_0_ represents the peak intensity at the onset of light stimulation. For bPGC experiments, F_0_ represents the intensity 1 sec after the onset of light stimulation.

### FlincG3 imaging in AFD

Animals transgenic for **gcy-8p**::*FlincG3* were grown at room temperature and shifted overnight to the desired cultivation temperature as young adults prior to imaging. Animals were placed on a pad of 10% agarose on a coverslip, together with 1 µl of a 1% solution of 0.1 µm polystyrene beads (Polysciences) (Kim *et al.* 2013). Animals were covered with a second glass coverslip and transferred to a glass slide on a Peltier system set to the desired starting temperature; 5 µl of glycerol was applied between the coverslips and glass slide to facilitate thermal conductivity. The temperature of the Peltier device was measured using a 15K thermistor (McShane), and controlled using LabView. FlincG3 fluorescence changes were measured in response to a 0.01 or 0.02°/cm linear thermal ramp, ranging from 13–18 17–21.5 or 19–22.5° for animals cultivated overnight at 15, 20 and 25°, respectively. Images were captured at 1 Hz with a 40× air objective (NA 0.9) using Metamorph software (Molecular Devices) and a digital camera (Orca; Hamamatsu). FlincG3 and RFP fluorescence were split using a DualView DV2 emission splitter. Changes in fluorescence intensity were quantified using custom MATLAB scripts (Takeishi *et al.* 2016). T*_AFD_ is defined as the temperature at which ΔF/F_0_ increased by >2% over >8 consecutive seconds with an average slope of >0.3% per second. The fluorescence intensity F−F_0_ (ΔF) at each time point was calculated by subtracting the fluorescence intensity at the dendrite (or cell body) from the background fluorescence intensity. The initial fluorescence intensity, F_0_, is the mean F−F_0_ of the first 10 images. Baseline fluorescence was set to zero to offset changes in fluorescence due to photobleaching or movement artifacts. Fluorescence changes were expressed as ΔF/F_0_ (%) ((F−F_0_)/F_0_ (%)).

### Thermosensory behavior

Worms were cultured overnight at 20° with food. Prior to the assay, 15 young adult worms were picked onto an unseeded 6 cm NGM plate to remove residual *Escherichia coli*. Animals were then transferred to an unseeded 10 cm NGM plate that was placed on an aluminum platform whose temperature was controlled using a Peltier system. The temperature of the plate was measured with a two-probe thermometer (Fluke electronics). To quantify negative thermotaxis, the temperature range on the plate was set from 23 to 28°, with a gradient steepness of 0.5°/cm. Animals were recorded at a rate of 1 Hz using a PixelLink CCD camera for 35 min. Positional data were acquired using WormLab software (MBF Bioscience), and analyzed using custom MATLAB scripts. Thermotaxis bias was defined as the ((total duration of movement or runs toward warmer temperatures) − (total duration of runs toward colder temperatures))/total run duration (Beverly *et al.* 2011).

### Imaging ASE and PHB

FlincG3 and jRGECO1a imaging were performed essentially as previously described for calcium imaging (Krzyzanowski *et al.* 2013). Briefly, for imaging ASER, day 1 adults grown at 20° were transferred from NGM plates containing OP50 to a 35 × 10 mm Petri dish containing chemotaxis buffer with 50 mM NaCl (25 mM K_3_PO_4_, pH 6.0, 1 mM CaCl_2_, 1 mM MgSO_4_, 50 mM NaCl, adjusted to 355 ± 2 mOsm with sorbitol); for ASEL, day 1 adults grown at 20° were transferred from NGM plates containing OP50 to a 35 × 10 mm Petri dish containing chemotaxis buffer with 0 mM NaCl (25 mM K_3_PO_4_, pH 6.0, 1 mM CaCl_2_, 1 mM MgSO_4_, adjusted to 355 ± 2 mOsm with sorbitol). The worms were then placed in a microfluidic device that can expose the animal to stimulus (Chronis *et al.* 2007). A Zeiss 40× air objective on an inverted microscope (Axiovert 200; Zeiss) was used for imaging, and images were taken at a rate of 1 Hz with a blue-light-exposure time of 30 msec, and a green-light-exposure time of 60 msec using an ORCA-Flash 2.8 camera (Hamamatsu) for a total of 103 frames for FlincG3 and jRGECO1a, respectively. Recordings were taken within 8 min of the animal’s exposure to chemotaxis buffer with either 50 mM NaCl for imaging ASER or 0 mM NaCl for imaging ASEL, and the animals were subjected to either ten 10-sec steps between chemotaxis buffer with 50 and 0 mM NaCl (each containing 1 mM levamisole (Sigma-Aldrich)) or switches between chemotaxis buffer with 50 mM NaCl for imaging ASER and 0 mM NaCl for imaging ASEL (each solution containing 1 mM levamisole (Sigma-Aldrich), and one solution containing fluorescein for at least three imaging days). Images were obtained using µManager (Version 1.4.22). Fluorescence intensity was measured using ImageJ. To calculate the fluorescence intensity at a given time point (F), the fluorescence intensity from the ROI encompassing the neuron was subtracted from the background ROI (outside of the animal). The fluorescence intensity F of the first three frames was averaged to calculate F_0_. We used ∆F/F_0_ (%) ((F–F_0_)/F_0_ (%)) to calculate the change in fluorescence intensity at a given time point.

To image FlincG3 in PHB neurons, animals were picked from NGM plates containing OP50 onto a Petri dish containing M13 control buffer, then placed tail-first into the microfluidic device. They were exposed to M13 control buffer for 15 sec, and then to 1 mM SDS in M13 for 15 sec; for switch control, they were exposed to M13 control buffer for 15 sec, then to M13 control buffer from a different channel for 15 sec. The animals were imaged at a rate of 2 Hz. To calculate the fluorescence intensity of PHB at each frame, ImageJ was used to measure the total intensity of the cell body. Background fluorescence was calculated by using ImageJ to measure the minimum pixel value in the area surrounding the cell body, and this pixel value was multiplied by the area of the cell body to get the total background. The total background was subtracted from the total intensity of the cell body to calculate the fluorescence intensity. The PHB FlincG3 fluorescence intensities were adjusted for photobleaching using the following method. The decrease in fluorescence during the first 29 frames when the animal was exposed to control buffer was presumed to be due to photobleaching. Therefore, the average difference between the values for the nth frame and the n+1th frame (up to the 29th frame) was calculated, and this average photobleaching value was then added back to each value in the series. F_0_ was the average of the response to buffer over the first three frames (1.5 sec) adjusted for photobleaching.

### NaCl cultivation assay

The NaCl cultivation assay was essentially performed as described (Kunitomo *et al.* 2013). Briefly, day 1 animals grown at 20° were transferred from OP50-containing NGM plates containing 50 mM NaCl to OP50-containing NGM plates containing 25, 50 or 100 mM NaCl for ∼6 hr before being placed on a chemotaxis assay plate containing regions of higher and lower NaCl for 45 min. Between 50 and 200 animals were placed onto the chemotaxis assay plates. Afterward, worms were stored in 4° for at least 16 hr before calculating the chemotaxis index. Chemotaxis index = [# animals at higher NaCl region − # animals at lower NaCl region]/[# animals at higher NaCl region + # animals at lower NaCl region + # animals outside origin].

### Behavioral assay response to the repellent SDS

SDS dry drop behavioral assays were conducted by using a hair pick to touch each worm on the nose to stimulate backward movement into a dry drop of 1 mM SDS in M13 buffer (Hilliard *et al.* 2002; Park *et al.* 2011). A dry drop is obtained by incubating an NGM plate overnight at 37° so that the SDS drop dries quickly into the plate, preventing wicking along the animal that might activate neurons in the head. An animal’s response time was defined as the amount of time it backed into the dry drop before terminating backward movement. The average response time to the dry drop of dilute SDS in M13 buffer was compared to the average response time to a drop of control M13 buffer. A response index was calculated by dividing the average response time to SDS by the average response time to M13 buffer. *nlp-1p*::*FlincG3*-expressing animals and *tax-4* mutants were each compared to wild-type animals assayed on the same day, and the wild-type response index was normalized to 100%. At least 80 animals of each genotype were tested: 40 for a response to M13, and 40 for a response to SDS.

### Muscle contraction assay

The muscle contraction assay was performed essentially as described (Gao *et al.* 2015). L4 animals were exposed to 0.9 mW/mm^2^ blue light (450–490 nm) for ∼20 sec, and relative body length was measured with a custom LabView script.

### Statistical analysis for ASE and PHB

Permutation tests were performed on the data using custom Python scripts. These permutation tests ask whether the differences between two populations are significant. Data from these two populations are randomly reshuffled into two populations. The data from one of the two reshuffled populations is then ranked relative to the data from one of the original two populations. Note that the “*n*” of the reshuffled data and the original data that are ranked must be equivalent. In our specific case, data were randomly reshuffled, and the resulting summated value of the randomly reshuffled data were calculated. The summated values of at least 500,000 randomly shuffled data were ranked relative to the summated value of the original, unshuffled data. The resulting rank of the summated value of the original data are the approximate (in the case of ranking a randomly selected subset of reshuffled data) or exact (in the case of ranking every possible combination of the data) *P*-value, which indicates the probability that the summated value of the original, unshuffled data were obtained by chance (Good 2006). The data measured reflect the chosen test statistic. The test statistics chosen for ASE recordings were slopes (for FlincG3) and response magnitudes (which were calculated by subtracting the maximum or minimum ΔF/F_0_ (%) for a 10 sec time range proceeding a switch from the value of ΔF/F_0_ (%) just before the switch; this was done for FlincG3 and jRGECO1a), and the test statistic chosen for PHB FlincG3 recordings was the area under the curve.

### Data availability statement

Strains and plasmids are available upon request. pSRW1JZ is available in Addgene (Plasmid # 129528). Supplemental files available at Figshare. File S1 contains supplemental materials and methods. Figure S1 through Figure S9 contains supplemental figures and associated figure legends. Supplemental material available at FigShare: https://doi.org/10.25386/genetics.8244758.

## Results

### FlincG3 is a circularly permuted GFP-based cGMP sensor

**Figure 1:**
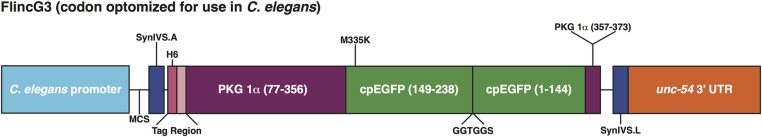
FlincG3 is a GFP-based cGMP sensor, which has been codon-optimized for use in *C. elegans*. FlincG3, which was initially characterized as a mammalian cGMP sensor, was codon-optimized for use in *C. elegans* (figure partially based on Bhargava *et al.* (2013)). This GFP-based sensor contains two in-tandem protein kinase G (PKG) I α cGMP binding domains that bind cGMP cooperatively (PKG1α (77–356); maroon); this regulatory PKG domain is attached to the N terminus of circularly permuted EGFP (cpEGFP; green). Changing the methionine at position 335, located outside the beta barrel of the cpEGFP domain, to lysine (M335K), improved the response amplitude of the sensor to cGMP (Bhargava *et al.* 2013). GGTGGS is a linker between the two GFP halves. This linker, along with the 6xHis-tag region (H6) and the Tag Region, were retained from the mammalian FlincG3 sensor. This *C. elegans* codon-optimized sensor, prepared by Genscript, was inserted into a worm-specific Fire vector, pPD95.75, which contains synthetic introns (SynIVS.A and SynIVS.L; blue) to facilitate expression, a multiple cloning site (MCS) and the 3′ untranslated region of *unc-54* (*unc-54* 3′ UTR; orange).

FlincG3 is a genetically encoded, circularly permuted GFP-based cGMP sensor that was initially characterized both *in vitro* and in cell lines (Bhargava *et al.* 2013). FlincG3 is based on FlincG, an earlier version of the sensor (Nausch *et al.* 2008). Like FlincG, FlincG3 contains the N-terminal region of protein kinase G (PKG) I α, which is comprised of two cGMP-binding domains that bind cGMP cooperatively. The first 77 amino acids of the N-terminal region of the cGMP-binding domain were deleted to prevent interactions with endogenous PKG (Nausch *et al.* 2008). This region of PKG I α is appended to the N-terminus of circularly permuted EGFP (cpEGFP) (Nausch *et al.* 2008; Bhargava *et al.* 2013). In the presence of cGMP, FlincG3 fluorescence increases, presumably due to the conformational changes of the sensor upon cGMP binding that allow the beta barrel of GFP to form and create the appropriate environment for fluorophore maturation (Nausch *et al.* 2008; Bhargava *et al.* 2013). The response amplitude of the FlincG3 sensor to cGMP was enhanced by a M335K substitution (analogous to the M153K substitution in GCaMP3) located outside the beta barrel of the cpEGFP domain (Figure 1) (Tian *et al.* 2009; Bhargava *et al.* 2013). FlincG exhibits rapid kinetics, and FlincG3 retains this property as it rapidly detects changes in endogenous cGMP levels in the nanomolar to low micromolar range in response to nitric oxide when expressed in HEK_GC/PDE5_ cells (Nausch *et al.* 2008; Bhargava *et al.* 2013). Additionally, FlincG3 fluorescence increases *in vitro* in response to a 230-fold lower concentration of cGMP than cAMP, suggesting that it preferentially binds to cGMP (Bhargava *et al.* 2013). For our study, we codon optimized FlincG3 for use in *C. elegans*, and inserted it into a standard *C. elegans* expression vector (Figure 1).

### Stimulation of blue-light-activated guanylyl cyclases increases FlincG3 fluorescence

**Figure 2:**
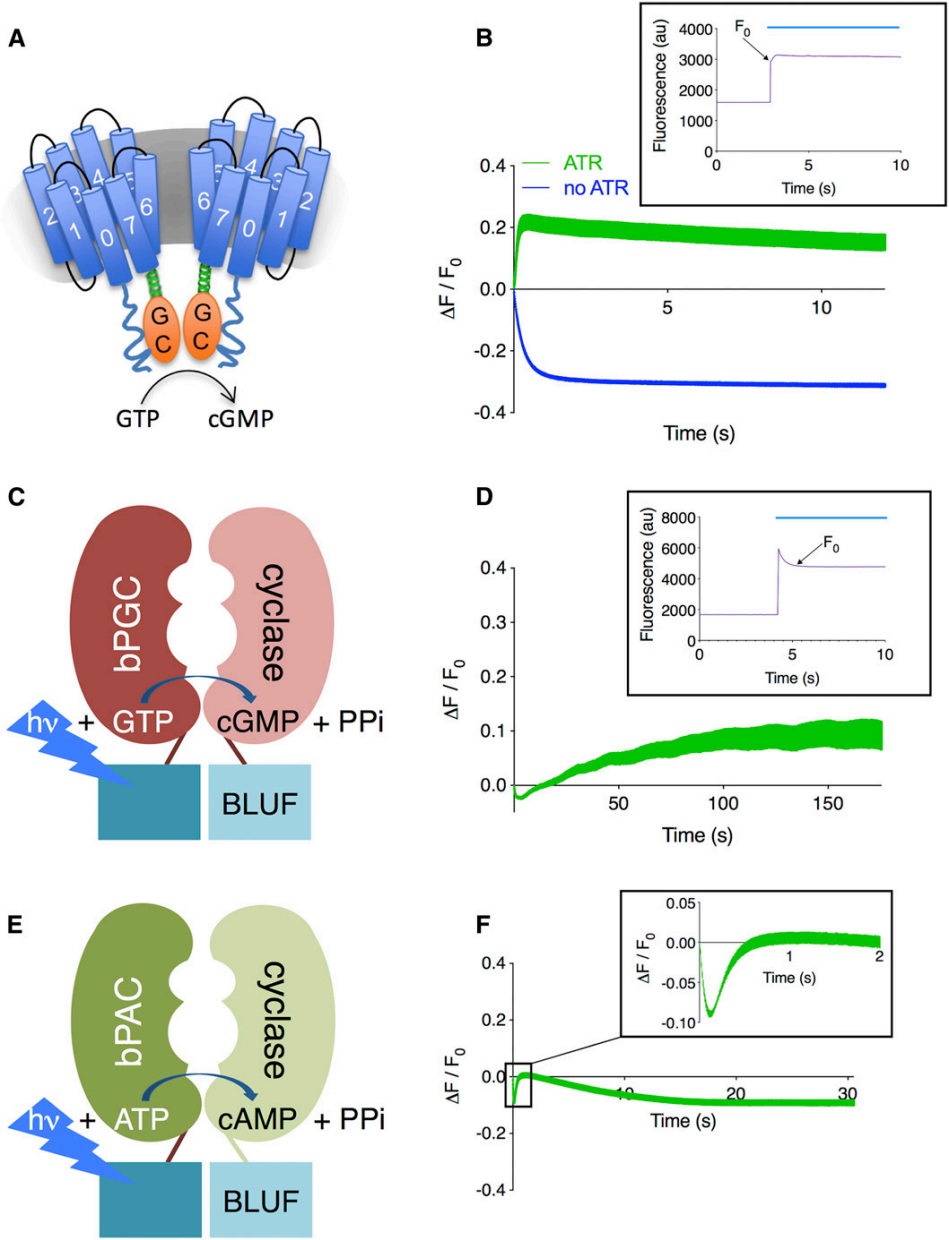
FlincG3 fluorescence increases upon stimulation of blue-light-activated guanylyl cyclases when coexpressed in body wall muscle cells. (A) BeCyclOp is a fungal blue-light-activated guanylyl cyclase that generates cGMP with a turnover rate of ∼17 cGMP per second at 20°; this figure is adapted from Figure 4A in Gao *et al.* (2015) under the Creative Commons Attribution License (https://creativecommons.org/licenses/by/4.0/). (B) ΔF/F_0_ for FlincG3 fluorescence intensity in *myo-3p*::CyclOp::SL2::mCherry; *myo-3p*::*FlincG3* animals grown in the absence and presence of all-*trans*-retinal (ATR). These animals express BeCyclOp and FlincG3 specifically in body wall muscle cells. *n* = 6 animals for FlincG3 fluorescence intensity without ATR (blue, bottom); *n* = 5 animals for FlincG3 fluorescence intensity with ATR (green, top). Inset shows the original traces with ATR and indicates F_0_; blue line indicates duration of blue light illumination. Traces are shown with SEM. (C) bPGC is a bacterial blue-light-activated guanylyl cyclase containing a BLUF (sensors of blue light using FAD) domain with an estimated turnover rate of 0.2 cGMP per second (Ryu *et al.* 2010). (D) ΔF/F_0_ for FlincG3 fluorescence intensity in *myo-3p*::bPGC::SL2::mCherry; *myo-3p*::*FlincG3* animals (*n* = 5 animals, 27 ROIs). These animals express bPGC and FlincG3 specifically in body wall muscle cells. Inset shows original traces and indicates F_0_; blue line indicates duration of blue light illumination. Traces are shown with SEM. (E) bPAC is a bacterial blue-light-activated adenylyl cyclase containing a BLUF (sensors of blue light using FAD) domain. *In vitro* cAMP production in the presence of blue light is 10 ± 2 nmol cAMP per minute per milligram (Stierl *et al.* 2011). (F) ΔF/F_0_ for FlincG3 fluorescence intensity in *myo-3p*::bPAC::SL2::mCherry, *myo-3p*::*FlincG3* animals. These animals express bPAC and FlincG3 specifically in body wall muscle cells. *n* = 7 animals. Inset shows the average of traces during the first 2 sec of recording. Traces are shown with SEM.

To test whether FlincG3 can detect rapid changes in cGMP levels in an intact animal, we utilized the *C. elegans* body wall muscle cells, which lack most endogenous GCs. We coexpressed the reporter along with heterologous light-inducible GCs that have different cGMP production rates (Ryu *et al.* 2010; Gao *et al.* 2015). BeCyclOp is a microbial rhodopsin from *Blastocladiella emersonii* that is linked to a cytosolic GC domain (Figure 2A). It detects photons by absorption using the retinal chromophore, and transmits this signal into activation of the GC domain (Gao *et al.* 2015). bPGC (*Beggiatoa sp*. photoactivated guanylyl cyclase) is a BLUF-domain photosensor that is coupled to a GC domain (Figure 2C). It originates from bPAC (*Beggiatoa sp*. photoactivated adenylyl cyclase) that was mutated to generate cGMP rather than cAMP (Ryu *et al.* 2010).

To test whether changes in FlincG3 fluorescence and dynamics correspond with cGMP production by BeCyclOp, animals coexpressing FlincG3 and BeCyclOp were grown with or without all-*trans*-retinal (ATR), which is required for BeCyclOp activity. When FlincG3 and BeCyclOp were coexpressed in body wall muscle cells in the presence of ATR, an acute increase in FlincG3 fluorescence (peak ΔF/F_0_ =0.218 ± 0.023 at 0.49 sec) was observed upon continuous blue light illumination, which activates BeCyclOp. This was followed by a slight decay over the duration of the recording (Figure 2B: top green trace). By contrast, animals grown without ATR and thus having no BeCyclOp activation exhibited an apparent decrease in FlincG3 fluorescence (ΔF/F_0_ plateaued at ∼0.310–0.312 beginning at 8.98 sec) when exposed to blue light (Figure 2B: bottom blue trace). The initial signal decayed; ΔF (F−F_0_) became negative, then plateaued and remained steady for the duration of the recording. Notably, the initial fluorescence intensity F_0_ (as measured in the absence of the rhodopsin cofactor ATR; bottom blue trace in Figure 2B) exhibited a rapid drop, possibly due to photoswitching behavior that was previously observed for other fluorescent proteins (Shaner *et al.* 2008; Akerboom *et al.* 2013). Taken together, we interpret these results to indicate that FlincG3 fluorescence correlates with the activation of BeCyclOp by blue light.

bPGC, a blue-light-activated GC derived from the corresponding adenylyl cyclase bPAC (also known as BlaC), produces 50-fold less cGMP per unit time relative to BeCyclOp (Ryu *et al.* 2010; Gao *et al.* 2015). FlincG3 fluorescence increased in the order of minutes upon continuous activation of bPGC with blue light (peak ΔF/F_0_ = 0.122 ± 0.023 at 145.2 sec) (Figure 2D). Note that at the onset of blue light illumination, FlincG3 fluorescence increased, then decreased rapidly (Figure 2D: inset); this is presumably the same rapid photoswitching observed in the BeCyclOp experiment (the time constants for decay of the signal were essentially identical: 0.383 sec for the no ATR trace in Figure 2B, and 0.297 sec for the trace in Figure 2D, in line with the hypothesis that this is due to the same photophysical process). We chose F_0_ after this photoswitching at 1 sec after light onset (Figure 2D: inset). At this time, meaningful amounts of cGMP begin to develop, as assessed from experiments in which bPGC was coexpressed in body wall muscle cells with the cyclic nucleotide-gated cation channel TAX-2/TAX-4; muscle contractions from ion influx begin to be observable after 1 sec of blue light exposure (Figure S1). After photoswitching, we observed a slow rise in FlincG3 fluorescence, which we interpret to be due to the slower kinetics of bPGC relative to BeCyclOp. By contrast, FlincG3 fluorescence increased acutely upon activation of BeCyclOp, suggesting that the rate of change of FlincG3 fluorescence correlates with the rate of cGMP production by each GC.

Bhargava *et al.* (2013) showed that FlincG3 has a 230-fold lower EC_50_ for cGMP relative to cAMP. To assess whether FlincG3 fluorescence changes with increasing cAMP levels *in vivo*, FlincG3 was coexpressed with bPAC, a bacterial blue-light-activated adenylyl cyclase, in body wall muscle cells (Figure 2E) (Stierl *et al.* 2011). Following a fast drop in fluorescence, these animals showed a 10% increase in FlincG3 fluorescence upon blue light stimulation of bPAC that peaked and decayed in a manner similar to that of the FlincG3 response to BeCyclOp, albeit with slightly slower kinetics (Figure 2F and Figure S2). Thus, FlincG3 appears to respond to cAMP. Indeed, bPAC is an efficient adenylyl cyclase that produces cAMP at the rate of 10 ± 2 nmol per minute per milligram (Stierl *et al.* 2011). Thus, it is not surprising that FlincG3 responds to the high production of cAMP by bPAC (Figure S2). Since there are no amino acid changes between mammalian and *C. elegans*-codon-optimized FlincG3, it is expected that the *C. elegans*-codon-optimized FlincG3 is also activated more effectively by cGMP relative to cAMP. However, these results indicate that it is important to control for the FlincG3 response to cAMP.

### FlincG3 fluorescence in the AFD thermosensory neuron endings is modulated by rising temperatures in an experience-dependent manner

**Figure 3:**
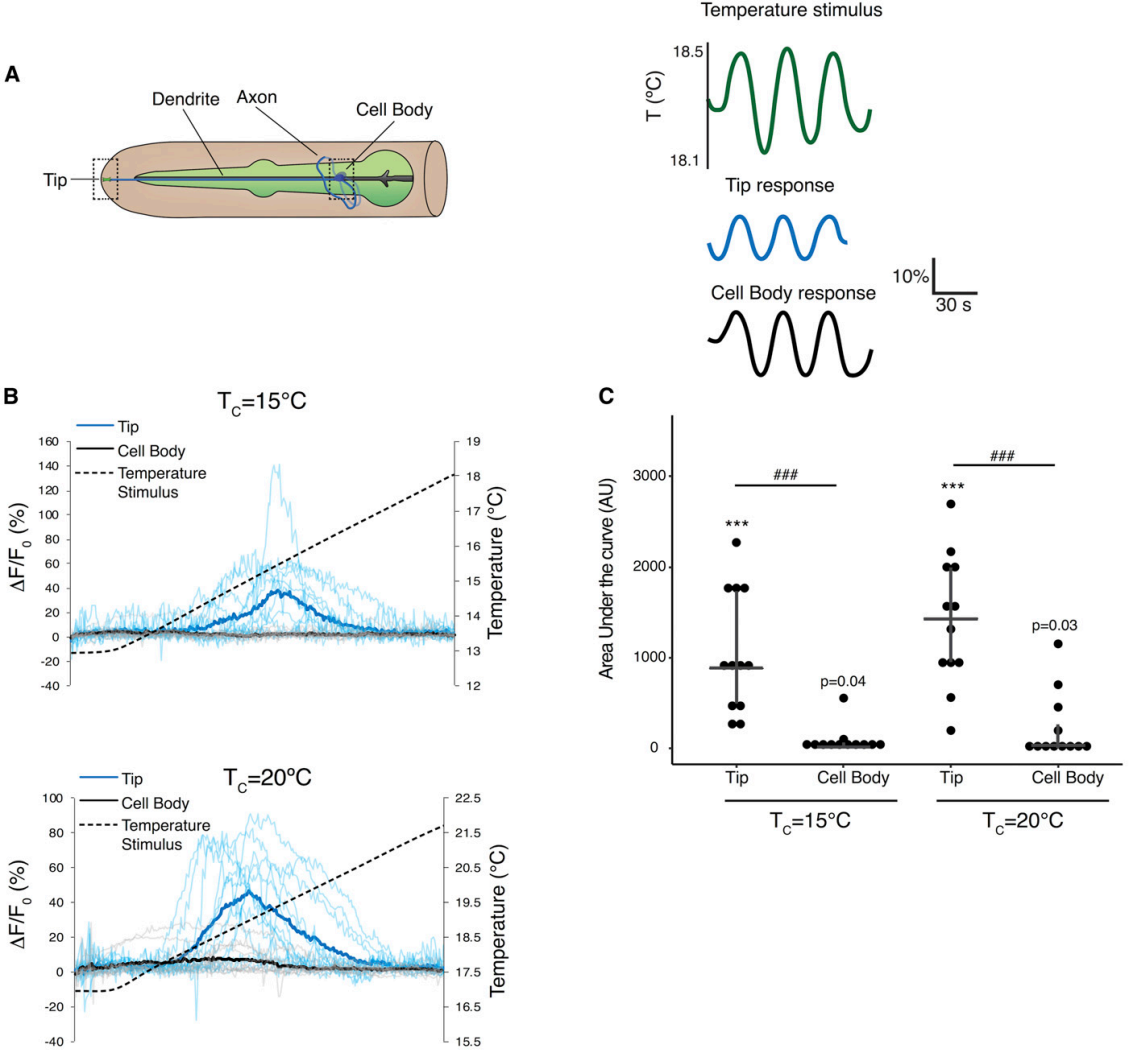
FlincG3 fluorescence at AFD thermosensory neuron endings changes in response to a rising temperature ramp in an experience-dependent manner. (A) (Left) Cartoon of the bilateral AFD thermosensory neurons in the head of *C. elegans* (adapted from www.wormatlas.org). Dashed boxes indicate regions in which imaging of FlincG3 fluorescence was performed. (Right) Schematic of oscillating temperature stimulus (green lines) and changes in intracellular calcium at the AFD sensory tips (blue lines) and cell bodies (black lines) measured via changes in fluorescence of the cameleon calcium sensor expressed in AFD (adapted from Clark *et al.* (2006)). (B) Changes in FlincG3 fluorescence at the tips (blue traces) and cell bodies (gray traces) of AFD neurons in response to a rising linear temperature ramp (black dashed lines). The slope of the ramp was 0.02° per second. Bolded blue and black traces indicate the average response, thinner traces indicate responses of individual neurons. Animals were cultivated overnight at temperature (*T_c_*) of 15° (top) or 20° (bottom). *n* = 12 animals for each T_c_. (C) Area under the curve measurements of the AFD neuron tip are different from measurements of the cell body. Horizontal bars indicate mean area under the curve, top and bottom of vertical bar indicates upper and lower quartile, respectively. *** indicates mean different from 0 at *P* < 0.0001 (mean *P*-value from bootstrap *t*-test). ### indicates tip different from cell body at *P* < 0.001 (two sample *t*-test).

The bilateral pair of AFD neurons are the primary thermosensors in *C. elegans* (Figure 3A) (Mori and Ohshima 1995). Environmental temperature changes are proposed to be transduced via modulation of intracellular cGMP levels in AFD (Goodman and Sengupta 2018). In the current model, rising temperatures are sensed by a family of rGCs to increase intracellular cGMP concentrations (Inada *et al.* 2006; Takeishi *et al.* 2016), which then gate the TAX-2/TAX-4 channel to modulate neuronal activity (Hedgecock and Russell 1975; Coburn and Bargmann 1996; Komatsu *et al.* 1996). In turn, cGMP-dependent PDEs hydrolyze cGMP to terminate signaling (Wang *et al.* 2013). Although temperature-regulated neuronal activity has been measured via quantification of thermoreceptor currents (Ramot *et al.* 2008) as well as changes in intracellular calcium levels (Kimura *et al.* 2004; Clark *et al.* 2006), changes in intracellular cGMP dynamics in response to temperature have not been previously directly visualized.

The thermosensor guanylyl cyclases and TAX-2/TAX-4 are localized specifically to the complex sensory endings of AFD (Inada *et al.* 2006; Nguyen *et al.* 2014). However, measurements of calcium dynamics using genetically encoded calcium indicators have shown robust calcium changes in response to temperature fluctuations both at the sensory endings of AFD as well as in their cell bodies, which is likely due to the amplification of the initial cGMP-driven signal via voltage-gated calcium channels (Figure 3A) (Kimura *et al.* 2004; Clark *et al.* 2006). To measure temperature-regulated cGMP dynamics, we generated a transgenic strain expressing FlincG3 specifically in AFD under the *gcy-8* promoter (Yu *et al.* 1997). On spatial thermal gradients, this strain exhibited robust AFD-mediated negative thermotaxis behavior (Hedgecock and Russell 1975; Mori and Ohshima 1995) (Figure S3A), indicating that AFD functions are not disrupted upon expression of FlincG3. We observed robust increases in FlincG3 fluorescence at the AFD sensory endings but not in the cell bodies in response to a rising temperature ramp (Figure 3, B and C and Figure S4), consistent with the production of a localized cGMP signal. The measured response was unlikely to be an artifact of animal movement, since ratiometric measurements performed with AFD-expressed RFP showed that the response correlated with FlincG3 but not RFP fluorescence changes (Figure S3B).

A key feature of AFD temperature responses is that the threshold of response (T*_AFD_) in this neuron type is closely correlated with the animal’s prior temperature experience (cultivation temperature, *T_c_*) (Kimura *et al.* 2004; Clark *et al.* 2006; Ramot *et al.* 2008; Kobayashi *et al.* 2016; Hawk *et al.* 2018). The mechanism underlying this temperature adaptation is unknown but has been proposed to be mediated via response adaptation of the TAX-2/TAX-4 channels, the thermosensor rGCs and/or the PDEs (Goodman and Sengupta 2018). Intriguingly, we found that T*_AFD_ of FlincG3-expressing cells was correlated with *T_c_* (Figure 3, B and C and Figure S4). This observation indicates that temperature adaptation occurs at the level of regulation of cGMP concentrations in AFD, suggesting that the rGCs and/or the PDEs are likely targets of adaptation in this neuron type. Together, these results confirm that temperature modulates cGMP levels in AFD in a temperature experience-dependent manner.

### The changes in FlincG3 and jRGECO1a fluorescence in response to NaCl concentration step changes are opposite in sign in the cell bodies of the gustatory neurons ASEL and ASER

Genetic and calcium imaging studies indirectly suggest that cGMP in the gustatory neurons ASEL and ASER mediates acute sensation of NaCl presentation and removal, respectively (Suzuki *et al.* 2008). ASEL and ASER express multiple rGCs asymmetrically and may use cGMP to gate a cyclic nucleotide-gated cation channel composed of TAX-2, TAX-4, and possibly CNG-4 (also known as CHE-6) upon changes in NaCl concentration (Suzuki *et al.* 2008; Ortiz *et al.* 2009; Smith *et al.* 2013). Consistent with this hypothesis, NaCl upsteps and downsteps trigger an influx of calcium into ASEL and ASER, respectively, and this calcium response was blocked in animals lacking TAX-2 or TAX-4 (Suzuki *et al.* 2008). Additionally, a study indicating that cGMP could be a putative second messenger in ASER revealed that loss of the rGC GCY-22 blunts chemotaxis to Cl^−^ (Smith *et al.* 2013). This suggests that cGMP levels could be modulated by changes in NaCl concentration (Ortiz *et al.* 2009). To explore this hypothesis, we coexpressed FlincG3 and the red calcium sensor jRGECO1a in the ASE neuron pair and monitored the sensors’ response in the ASEL and ASER cell bodies to ten 10-sec steps between 50 and 0 mM NaCl (Dana *et al.* 2016).

**Figure 4:**
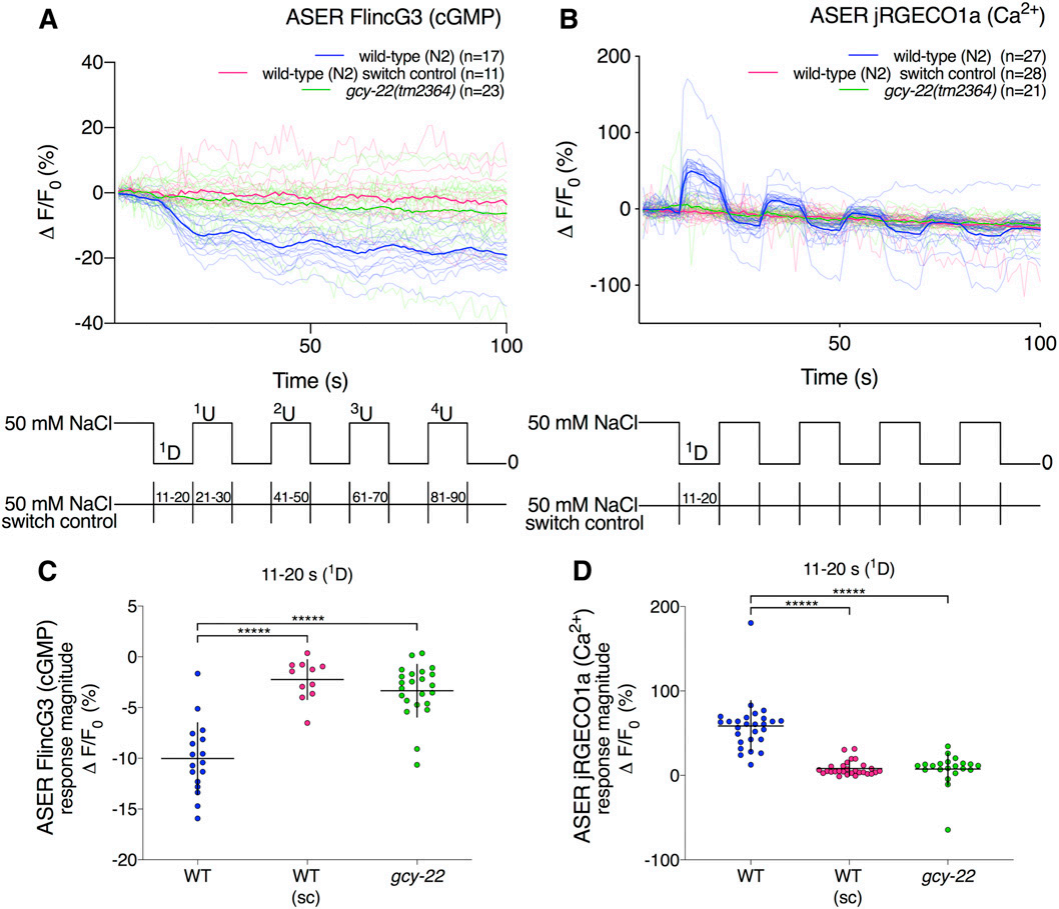
FlincG3 and jRGECO1a fluorescence in the ASER cell body are opposite in sign in response to 50 mM NaCl step changes and depend on the receptor guanylyl cyclase GCY-22. (A) Average fluorescence response (ΔF/F_0_ (%)) of FlincG3 in the ASER cell body shown as bolded traces responding to either ten 10-sec steps between 50 and 0 mM NaCl or switch control (represented at the bottom of the panel). Thinner traces are from individual recordings for each condition. ASER FlincG3 fluorescence in wild-type (N2) animals decreases in response to a 50–0 mM NaCl downstep (blue traces). These responses are not seen in *gcy-22**(**tm2364**)* animals (green traces) or when wild-type animals are exposed to switch control (pink traces). *n* = 17, *n* = 23, and *n* = 11 animals for wild type, *gcy-22**(**tm2364**)* and wild-type switch control, respectively. (B) Average fluorescence response (ΔF/F_0_ (%)) of jRGECO1a in the ASER cell body shown as bolded traces responding to either ten 10-sec steps between 50 and 0 mM NaCl or switch control (represented at the bottom of the panel). Thinner traces are from individual recordings for each condition. ASER jRGECO1a fluorescence in wild-type (N2) animals increases in response to a 50–0 mM NaCl downstep (blue traces). On average, these responses are not seen in *gcy-22**(**tm2364**)* animals (green traces) or when wild-type animals are exposed to switch control (pink traces). *n* = 27, *n* = 21, and *n* = 28 animals for wild-type, *gcy-22**(**tm2364**)*, and wild-type switch control, respectively. (C) FlincG3 fluorescence in the ASER cell body decreases in response to a 50–0 mM NaCl downstep in wild-type animals. The response magnitudes for the first 50–0 mM NaCl downstep between wild-type and *gcy-22**(**tm2364**)* animals are different (*n* = 17 (first set, blue; wild-type), *n* = 23 (third set, green; *gcy-22*); permutation test *P* < 0.00001). In wild-type animals, the response magnitudes for the first 50–0 mM NaCl downstep are also different from those of the switch control (*n* = 17 (first set, blue; wild-type), *n* = 11 (second set, pink; switch control); permutation test *P* < 0.00001). Individual dots are the response magnitudes calculated for each animal. sc, switch control. Horizontal bars indicate mean; vertical error bars indicate ± SD. See *Materials and Methods* for details of statistical analysis. (D) jRGECO1a fluorescence in the ASER cell body increases in response to a 50–0 mM NaCl downstep in wild-type animals. The response magnitudes for the first 50–0 mM NaCl downstep between wild-type and *gcy-22**(**tm2364**)* animals are different (*n* = 27 (first set, blue; wild-type), *n* = 21 (third set, green; *gcy-22*); permutation test *P* < 0.00001). In wild-type animals, the response magnitudes for the first 50–0 mM NaCl downstep are also different from those of the switch control (*n* = 27 (first set, blue; wild-type), *n* = 28 (second set, pink; switch control); permutation test *P* < 0.00001). Individual dots are the response magnitudes calculated for each animal. sc, switch control. Horizontal bars indicate mean; vertical error bars indicate ± SD. See *Materials and Methods* for details of statistical analysis.

In the ASER cell body, FlincG3 fluorescence decreased in response to a 50–0 mM NaCl downstep and stopped decreasing in response to the first 0–50 mM NaCl upstep (Figure 4A: blue traces). To test whether changes in ASER FlincG3 fluorescence were due to changing NaCl concentrations or to the potential fluctuation in pressure due to the change in flow of the stimulus presentation stream, we examined the sensor’s responses to ten 10-sec switches of 50 mM NaCl (Figure 4A: key at bottom of the panel). ASER FlincG3 fluorescence did not change in these animals in response to switching (Figure 4A: pink traces). The response magnitudes of the first downstep between wild-type animals and wild-type switch control animals are different, suggesting that FlincG3 responds to the decrease in NaCl concentration in wild-type animals (Figure 4C: first set, blue; wild type and second set, pink; switch control, *P* < 0.00001; see *Materials and Methods* for statistical analysis). Additionally, the slopes of the first downstep between animals recorded in response to changing NaCl concentration and those recorded in response to simply switching the buffer stream are different (Figure S5A: first set, blue; wild type and third set, pink; switch control, *P* < 0.00001; see *Materials and Methods* for statistical analysis). Together, this suggests that ASER FlincG3 fluorescence changes were due to NaCl concentration steps and not due to fluctuations in fluid pressure on the exposed nose of the animal. We also found that the slopes between the first downstep and upstep are different in wild-type animals (*P* < 0.00001; see *Materials and Methods* for statistical analysis), suggesting that FlincG3 responds quickly to changing NaCl concentrations (Figure S5A: first pair, blue).

Using our FlincG3 and jRGECO1a coexpressing line (Figure 4), we observed an increase in jRGECO1a fluorescence in the ASER cell body in response to NaCl downsteps, as was previously reported for lines that only express a calcium reporter (Figure 4B: blue traces) (Suzuki *et al.* 2008). As with FlincG3, we examined jRGECO1a’s responses to ten 10-sec switches of 50 mM NaCl (Figure 4B: key at bottom of the panel). ASER jRGECO1a fluorescence did not appreciably change in these animals in response to switching (Figure 4B: pink traces). The ASER jRGECO1a response magnitudes of the first downstep are different between wild-type animals exposed to a decrease in NaCl concentration and wild-type animals exposed to switch control (Figure 4D: first set, blue; wild type and second set, pink; switch control, *P* < 0.00001; see *Materials and Methods* for statistical analysis). This suggests that jRGECO1a in ASER responds to decreases in NaCl concentration in wild-type animals coexpressing FlincG3.

FlincG3 has a cGMP-binding motif that could also potentially accommodate cAMP, albeit with lower affinity (Nausch *et al.* 2008; Bhargava *et al.* 2013). To assess whether ASER FlincG3 fluorescence changes were dependent on cGMP or cAMP, we recorded ASER FlincG3 fluorescence in animals lacking the rGC GCY-22. Though other rGCs are expressed in ASER (Ortiz *et al.* 2006, 2009; Kunitomo *et al.* 2013), loss of GCY-22 produces the most severe behavioral defects in Cl^−^ and NaCl chemotaxis (Ortiz *et al.* 2009; Kunitomo *et al.* 2013). Consistent with these findings, and, in contrast to wild-type animals, ASER FlincG3 fluorescence did not change in *gcy-22**(**tm2364**)* animals in response to NaCl downsteps or upsteps (Figure 4A: green traces). Furthermore, ASER jRGECO1a fluorescence changes were diminished in *gcy-22* animals in response to NaCl downsteps and upsteps (Figure 4B: green traces). The response magnitudes of the first downstep differ between wild-type animals and *gcy-22**(**tm2364**)* animals with respect to both FlincG3 (Figure 4C: first set, blue; wild type and third set, green; *gcy-22*, *P* < 0.00001; see *Materials and Methods* for statistical analysis) and jRGECO1a (Figure 4D: first set, blue; wild type and third set, green; *gcy-22*, *P* < 0.00001; see *Materials and Methods* for statistical analysis). Additionally, the slopes of ASER FlincG3 fluorescence for the first downstep between wild-type animals and *gcy-22**(**tm2364**)* animals are different (Figure S5A: first set, blue; wild type and fifth set, green; *gcy-22*, *P* < 0.00001; see *Materials and Methods* for statistical analysis). Together, these findings indicate that (1) changes in ASER FlincG3 fluorescence require GCY-22 and likely result from changes in cGMP rather than cAMP; and (2) changes in ASER jRGECO1a fluorescence also, to a large extent, require GCY-22.

FlincG3 fluorescence in ASER also increased in wild-type animals in response to the second, third, and fourth 0–50 mM NaCl upstep (Figure S5B). This was dependent on both an actual change in NaCl concentration (Figure S5B: compare blue set with pink set in each group) and the rGC GCY-22 (Figure S5B: compare blue set with green set in each group). Together, these results suggest that FlincG3 can report rapidly changing increases and decreases in endogenous cGMP levels in ASER.

**Figure 5:**
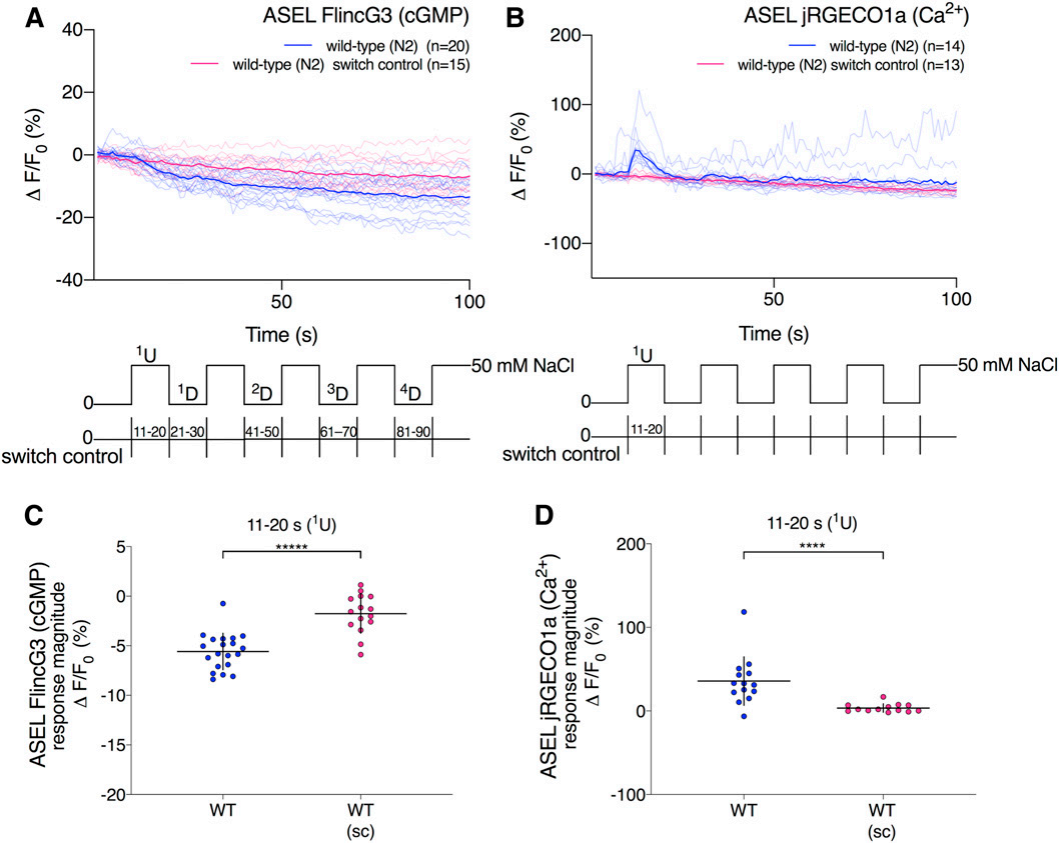
FlincG3 and jRGECO1a fluorescence in the ASEL cell body are opposite in sign in response to a 0–50 mM NaCl upstep. (A) Average fluorescence response (ΔF/F_0_ (%)) of FlincG3 in the ASEL cell body shown as bolded traces responding to either ten 10-sec steps between 0 and 50 mM NaCl or switch control (represented at the bottom of the panel). Thinner traces are from individual recordings for each condition. ASEL FlincG3 fluorescence in wild-type (N2) animals decreases in response to a 0–50 mM NaCl upstep (blue traces). These responses are not seen in wild-type animals exposed to switch control (pink traces). *n* = 20 and *n* = 15 animals for wild-type and wild-type switch control, respectively. (B) Average fluorescence response (ΔF/F_0_ (%)) of jRGECO1a in the ASEL cell body shown as bolded traces responding to either ten 10-sec steps between 0 and 50 mM NaCl or switch control (represented at the bottom of the panel). Thinner traces are from individual recordings for each condition. ASEL jRGECO1a fluorescence in wild-type (N2) animals increases in response to a 0–50 mM NaCl upstep (blue traces). These responses are not seen when wild-type animals are exposed to switch control (pink traces). *n* = 14 and *n* = 13 animals for wild-type and wild-type switch control, respectively. (C) FlincG3 fluorescence in the ASEL cell body decreases in response to a 0–50 mM NaCl upstep in wild-type animals. The response magnitudes for the first 0–50 mM NaCl upstep between wild type and wild-type switch control animals are different (*n* = 14 (first set, blue; wild type), *n* = 13 (second set, pink; switch control); permutation test *P* < 0.00001). Individual dots are the response magnitudes calculated for each animal. sc, switch control. Horizontal bars indicate mean; vertical error bars indicate ± SD. See *Materials and Methods* for details of statistical analysis. (D) jRGECO1a fluorescence in the ASEL cell body increases in response to a 0–50 mM NaCl upstep in wild-type animals. The response magnitudes for the first 0–50 mM NaCl upstep between wild type and wild-type switch control animals are different (*n* = 14 (first set, blue; wild type), *n* = 13 (second set, pink; switch control); permutation test *P* < 0.0001). Individual dots are the response magnitudes calculated for each animal. sc, switch control. Horizontal bars indicate mean; vertical error bars indicate ± SD. See *Materials and Methods* for details of statistical analysis.

To test whether ASEL FlincG3 fluorescence also changed in response to NaCl concentration step changes, we performed ten 10-sec steps between 0 and 50 mM NaCl (Figure 5A: key at bottom of the panel). In the ASEL cell body, FlincG3 fluorescence decreased in response to the first 0–50 mM NaCl upstep (Figure 5A: blue traces). To test whether changes in ASEL FlincG3 fluorescence were due to the 0–50 mM NaCl upstep or to the potential fluctuation in pressure due to the change in flow of the stimulus presentation stream, we examined the sensor’s responses to ten 10-sec switches of 0 mM NaCl (Figure 5A: key at bottom of the panel). ASEL FlincG3 fluorescence did not change in these animals in response to switching (Figure 5A: pink traces), and the ASEL FlincG3 response magnitudes of wild-type animals exposed to the first NaCl upstep differed from the ASEL FlincG3 response magnitudes of wild-type animals exposed to switch control (Figure 5C: first set, blue; wild type and second set, pink; switch control, *P* < 0.00001; see *Materials and Methods* for statistical analysis). Additionally, the slopes of ASEL FlincG3 fluorescence of wild-type animals exposed to the first NaCl concentration upstep differed from the slopes of ASEL FlincG3 fluorescence of wild-type animals exposed to switch control (Figure S6A: first set, blue; wild type and third set, pink; switch control, *P* < 0.00001; see *Materials and Methods* for statistical analysis). We also found that the slopes of ASEL FlincG3 fluorescence between the first upstep and downstep are different in wild-type animals (Figure S6A: first pair, blue, *P* < 0.00001; see *Materials and Methods* for statistical analysis). In contrast, the slopes of ASEL FlincG3 fluorescence are not different in wild-type animals exposed to switch control (Figure S6A: second pair, pink, *P* ns; see *Materials and Methods* for statistical analysis). Together, this suggests that the ASEL FlincG3 fluorescence change was due to the NaCl concentration upstep, and not to fluctuations in fluid pressure on the exposed nose of the animal.

In contrast to FlincG3, jRGECO1a fluorescence increased in the ASEL cell body in response to the first NaCl upstep, as was previously reported (Figure 5B: blue traces) (Suzuki *et al.* 2008). As with FlincG3, we examined ASEL jRGECO1a’s responses to ten 10-sec switches of 0 mM NaCl (Figure 5B: key at bottom of the panel). ASEL jRGECO1a fluorescence did not change in these animals in response to switching (Figure 5B: pink traces). The ASEL jRGECO1a response magnitudes of the first upstep are different between wild-type animals exposed to an increase in NaCl concentration and wild-type animals exposed to switch control (Figure 5D: first set, blue; wild type and second set, pink; switch control, *P* < 0.0001; see *Materials and Methods* for statistical analysis). This suggests that jRGECO1a in ASEL is responding to the first 0–50 mM NaCl upstep in wild-type animals.

In contrast to ASER FlincG3 fluorescence in response to the second and fourth 0–50 mM NaCl upstep, wild-type ASEL FlincG3 fluorescence did not change relative to switch control in response to the second and fourth 50–0 mM NaCl downstep (Figure S6B). These results suggest that FlincG3 fluorescence in ASEL, in contrast to ASER, does not necessarily change in response to repeatedly changing NaCl concentrations. This appears to be consistent with jRGECO1a fluorescence in ASEL, which also does not seem to change in response to repeatedly changing NaCl concentrations (Figure 5B: blue traces).

### Animals expressing FlincG3 in the ASE neuron pair prefer higher NaCl concentrations relative to animals that do not express the reporter

**Figure 6:**
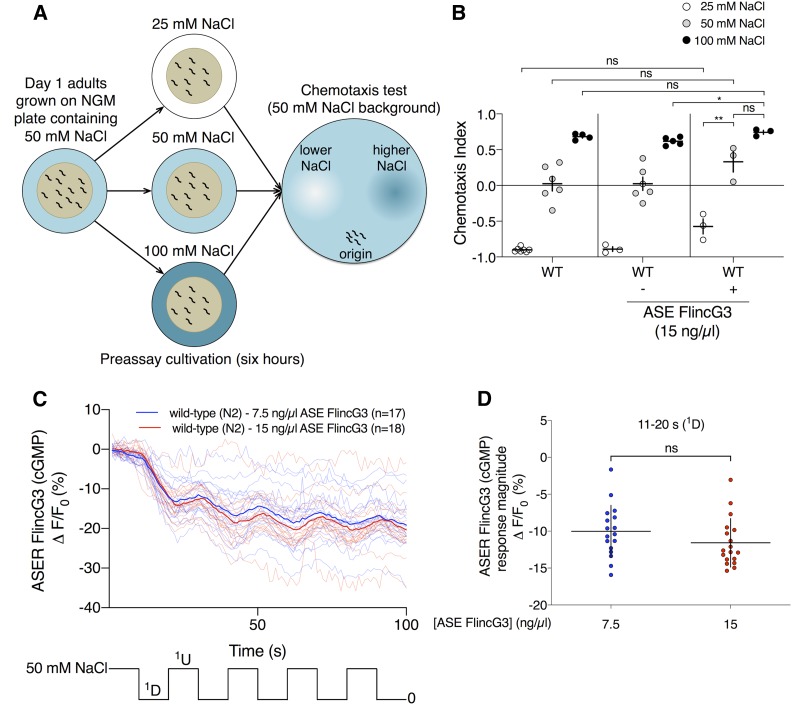
FlincG3 expression in ASE does not affect reporter activity but can increase NaCl seeking behavior. (A) Animals were tested for NaCl cultivation preference (figure based on Kunitomo *et al.* (2013)). Briefly, animals were placed on cultivation plates with various NaCl concentrations for ∼6 hr before being placed on an assay plate with regions of higher and lower NaCl concentration. (B) Wild-type animals injected with 15 ng/µl ASE FlincG3 (*flp-6p*::*FlincG3*) exhibited a preference for higher NaCl concentration while maintaining behavioral plasticity; animals from this line were recorded and analyzed for (C and D). Wild-type animals cultivated at 25 mM NaCl, 50 mM NaCl, and 100 mM NaCl have a chemotaxis index (CI) approaching −1, 0, and 0.75, respectively (first set). The line injected with 15 ng/µl ASE FlincG3 produces two types of progeny: those that do not express the array (nontransgenic siblings) and those that do (transgenic siblings). Nontransgenic siblings behave like wild-type animals (second set; Welch’s *t*-test, ns). Transgenic animals cultivated at each NaCl concentration exhibit a slight but not significant preference for higher NaCl concentration relative to wild-type animals (third set; Welch’s *t*-test, ns). Individual dots represent a single trial. Horizontal bars indicate mean; vertical error bars indicate ± SEM. (C) Average fluorescence response (ΔF/F_0_ (%)) of FlincG3 in the ASER cell body shown as bolded traces responding to ten 10-sec steps between 50 and 0 mM NaCl (represented at the bottom of the panel). Thinner traces are from individual recordings for each condition. ASER FlincG3 fluorescence in wild-type (N2) animals decreases in response to a 50–0 mM NaCl downstep; this is seen in animals injected with either 7.5 ng/µl (blue traces) or 15 ng/µl (red traces) ASE FlincG3. *n* = 18 and *n* = 17 wild-type animals injected with 15 ng/µl ASE FlincG3 and 7.5 ng/µl ASE FlincG3, respectively. The data for the strain injected with 7.5 ng/µl ASE FlincG3 are the same data that were used in Figure 4A as wild type (N2). (D) The response magnitudes of the first 50–0 mM NaCl downstep are not different between wild-type animals injected with 7.5 ng/µl ASE FlincG3 (*n* = 17; first set, blue) and wild-type animals injected with 15 ng/µl ASE FlincG3 (*n* = 18; second set, red) (permutation test ns). The data for the strain injected with 7.5 ng/µl ASE FlincG3 are the same as the data used in Figure 4C as wild type, first set, blue. Individual dots are the response magnitudes calculated for each animal. Horizontal bars indicate mean; vertical error bars indicate ± SD. See *Materials and Methods* for details of statistical analysis.

*C. elegans* requires ASER activity to adjust their preferred NaCl concentration to the concentration at which they were last fed; if ASER is killed, the animal’s movement is less directed in response to a linear NaCl gradient (Luo *et al.* 2014). Plasticity requires NaCl sensation, which in turn requires cGMP signaling; thus it is not surprising that *gcy-22**(**tm2364**)* animals, which do not respond to NaCl concentration changes in ASER (Figure 4), do not exhibit a preference for the concentration of NaCl at which they were cultivated (Kunitomo *et al.* 2013). To assess whether ASE FlincG3 expression affected an animal’s ability to exhibit a preference for its cultivation NaCl concentration, the behavior of ASE FlincG3-expressing wild-type animals was compared to their nontransgenic siblings that did not express the ASE FlincG3 array and wild-type animals. Animals were cultivated for ∼6 hr in the presence of OP50 *E. coli* bacteria on an NGM plate containing 25, 50, or 100 mM NaCl, then placed onto a chemotaxis assay plate containing a NaCl gradient from ∼40–90 mM NaCl (Figure 6A, based on Kunitomo *et al.* (2013)). A chemotaxis index (CI) of 1 indicates the animals’ preference for the higher NaCl concentration, and a CI of –1 indicates the animals’ preference for the lower NaCl concentration. Wild-type and nontransgenic siblings behaved as previously described; animals that were cultivated at 25, 50, and 100 mM NaCl had a CI approaching −1, 0, and 0.75, respectively (Figure 6B: first and second set of data points, respectively) (Kunitomo *et al.* 2013). The ASE FlincG3-expressing animals’ NaCl concentration preference at each cultivation NaCl concentration was higher, though not significantly different from wild-type animals (Figure 6B: third set of data points); however, their preference for a higher NaCl concentration was significantly different from their nontransgenic siblings only when they were cultivated at 100 mM NaCl (*P* < 0.05; Welch’s *t*-test). Additionally, the animals’ preference for a higher NaCl concentration seemed different from their nontransgenic siblings when they were cultivated at 50 mM NaCl, though this was not significant (*P* = 0.15; Welch’s *t*-test). This difference is presumably due to the variability in the transgenic animals’ chemotaxis responses to NaCl when cultivated at 50 mM NaCl. Other lines, injected with the same concentration of ASE FlincG3, exhibited NaCl seeking behavior that was significantly different from both wild-type animals and their nontransgenic siblings (Figure S7). This may indicate that FlincG3 expression lowers free cGMP levels and therefore interferes with an aspect of cGMP dynamics in ASER that is required for food to reset the animals’ preference to their cultivation NaCl concentration.

ASER FlincG3 fluorescence was recorded for the line that exhibited behavior closest to that of the wild-type animals (see Figure 6B). Importantly, in these animals, ASER FlincG3 fluorescence decreased in response to a 50–0 mM NaCl downstep, and stopped decreasing in response to a 0–50 mM NaCl upstep, with the slopes between the first downstep and upstep being different (Figure 6C: red traces; Figure S8: second pair, red; *P* < 0.00001; see *Materials and Methods* for statistical analysis). This finding is similar to that observed with ASE FlincG3 injected at a lower concentration (Figure 6C: blue traces and Figure S8: first pair, blue; note that these data points are reproduced from Figure 4A: blue traces and Figure S5A: first pair, blue, respectively). Furthermore, the ASER FlincG3 response magnitudes and slopes of the first downstep of wild-type animals injected at a lower *vs.* higher concentration are not different, indicating that the concentrations injected did not influence recordings (Figure 6D: compare first blue set (note that these data points are reproduced from Figure 4C: first set, blue) with second red set; *P* ns and Figure S8: compare first blue set (note that these data points are reproduced from Figure S5A: first set, blue) with third red set; *P* ns; see *Materials and Methods* for statistical analysis). Thus, though FlincG3 reliably reports the stimulus-induced changes in the gustatory sensory neuron ASER, its expression may subtly alter behavior, and this must be controlled for by comparing the behavior of transgenic lines with their nontransgenic siblings.

### FlincG3 fluorescence increases in PHB chemosensory tail neurons in response to SDS

**Figure 7:**
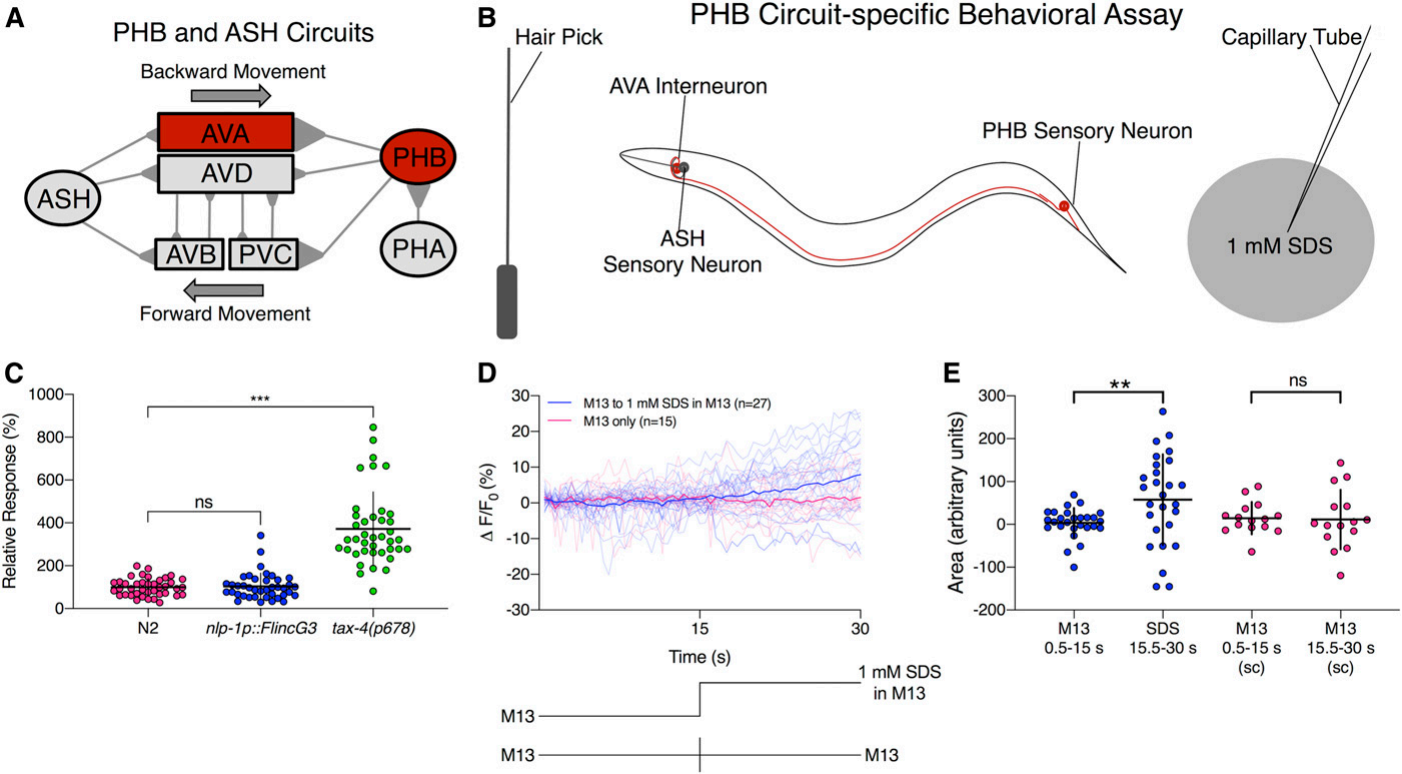
FlincG3 allows visualization of cGMP production in PHB chemosensory neurons in response to the repellent SDS. (A) Diagram of the PHB circuit; this figure is reproduced from Figure 1C in Varshney *et al.* (2018) under the Creative Commons Attribution License (https://creativecommons.org/licenses/by/4.0/). The primary postsynaptic partners of PHB neurons are the AVA backward command interneurons and the PVC forward command interneurons (Hilliard *et al.* 2002; Park *et al.* 2011; Tran *et al.* 2017). (B) Diagram of the sodium dodecyl sulfate (SDS) behavioral assay, which tests the PHB circuit response to 1 mM SDS; this figure is reproduced from Figure 1D in Varshney *et al.* (2018) under the Creative Commons Attribution License (https://creativecommons.org/licenses/by/4.0/). Animals are induced to move backward with a nose touch from a hair pick (Hilliard *et al.* 2002; Park *et al.* 2011; Tran *et al.* 2017). A drop of 1 mM SDS is placed behind them on a dry NGM plate, so that the drop quickly absorbs into the media, preventing wicking along the worm. The time that the animal backs into the drop before stopping is measured. (C) FlincG3 expression in PHB does not affect the response of animals to SDS (*P* ns). For reference, loss of *tax-4* causes a severe defect in the ability to sense SDS (Hilliard *et al.* 2002). One-factor ANOVA analysis was first performed, then two-sample *t*-tests and the Hochberg procedure for multiple comparisons were performed. (D) When FlincG3 is expressed in PHB neurons, ΔF/F_0_ (%) increases steadily after introduction of 1 mM SDS. Average fluorescence response (ΔF/F_0_ (%)) of FlincG3 in the PHB cell body shown as bolded traces responding to either 1 mM SDS presentation or M13 buffer alone (represented at the bottom of the panel). Thinner traces are from individual recordings for each condition. *n* = 15 for animals exposed to M13 control buffer, then switched to another channel with M13 control buffer, and *n* = 27 for animals exposed to M13 control buffer, then switched to another channel with 1 mM SDS in M13 buffer. (E) The areas under the curve for recordings before and after SDS presentation are different (permutation test, *P* < 0.01). The areas under the curve for M13-only recordings are not different (permutation test, *P* ns). Horizontal bars indicate mean; vertical error bars indicate ± SD. See *Materials and Methods* for details of statistical analysis.

To examine the ability of FlincG3 to report cGMP changes in a neuron with a third modality, we expressed FlincG3 in the nociceptive PHB neurons that had been predicted to use cGMP as a second messenger. The PHBs are a pair of bilaterally symmetric sensory neurons located in the lumbar ganglia that extend ciliated dendrites into the phasmid structures within the tail of *C. elegans*. PHB neurons are chemosensory cells that are required for the avoidance of noxious chemicals such as SDS, dodecanoic acid, and other cues (Figure 7A) (Hilliard *et al.* 2002; Park *et al.* 2011; Tran *et al.* 2017; Zou *et al.* 2017). TAX-4 is required for PHB-mediated SDS avoidance (Figure 7C), and calcium imaging has shown that PHB responds to SDS (Zou *et al.* 2017). This suggests that PHB may exhibit changes in cGMP levels in response to SDS that could be monitored by recording changes in FlincG3 fluorescence.

To test whether FlincG3 affects the function of the PHB circuit, SDS response assays (Figure 7B) were performed on wild-type animals and animals expressing FlincG3 in PHB neurons. On average, wild-type animals halt movement into a drop of 1 mM SDS in <1 sec (Figure S9). If PHB function is impaired, as in *tax-4* mutants, animals continue moving into a drop of SDS as if it were a control buffer (M13) (Figure S9); this increases the relative response index to ∼300% (Figure 7C). We found that the aversive response to SDS is unaffected by PHB FlincG3 expression, indicating that FlincG3 does not affect PHB function (Figure 7C and Figure S9).

To determine if cGMP changes in PHB neurons could be detected using FlincG3, the sensor’s fluorescence in the cell body was measured in animals that were first exposed to control buffer (M13), then to 1 mM SDS in M13 buffer. PHB FlincG3 fluorescence remained largely steady in the absence of 1 mM SDS, but began to increase linearly (*R*^2^ =0.95) upon exposure to 1 mM SDS (Figure 7D). To test whether changes in PHB FlincG3 fluorescence were due to 1 mM SDS exposure or to the potential fluctuation in pressure due to the change in flow of the stimulus presentation stream, we examined the sensor’s response to an M13 to M13 control switch (Figure 7D: key at bottom of the panel). We found that PHB FlincG3 fluorescence did not increase when animals were exposed only to M13 buffer for the duration of the recording (Figure 7D). The areas under the curve before and after SDS presentation were significantly different (*P* < 0.01; see *Materials and Methods* for statistical analysis) (Figure 7E: first pair, blue). In contrast, the areas under the curve for M13-only recordings were not significantly different (*P* ns; see *Materials and Methods* for statistical analysis) (Figure 7E: second pair, pink). This suggests that cGMP increases in response to SDS and that FlincG3 responds acutely to endogenously produced cGMP that is induced by an external stimulus (Figure 7, D and E).

## Discussion

### FlincG3 can be used as a sensor for cGMP dynamics in *C. elegans*

The GFP-based cGMP sensor FlincG3 was used successfully to monitor the dynamics of this second messenger in a number of cells in *C. elegans*. First, FlincG3 was used to monitor the kinetics of cGMP production in body wall muscle cells, which lack most endogenous GCs. The rate of increase in FlincG3 fluorescence corresponded with the rate of cGMP produced by coexpressed blue-light-activated GCs. FlincG3 fluorescence increased within <1 sec of activation of BeCyclOp, which produces 17 cGMP molecules per second. In contrast, FlincG3 fluorescence increased in the order of minutes upon activation of bPGC, which produces cGMP at a 50-fold lower rate relative to BeCyclOp (Gao *et al.* 2015). FlincG3 fluorescence slightly increased in the presence of cAMP in *C. elegans* in response to activation of bPAC. Thus, care must be taken to control for fluctuations in cAMP by imaging in backgrounds that lack cGMP production. The high rate of cAMP production due to bPAC, however, is likely exceeding any intrinsic cAMP production by several fold, thus side effects from intrinsic cAMP fluctuation may affect cGMP imaging only to a minor extent.

### FlincG3 reveals cGMP dynamics in sensory neurons that use cGMP as a second messenger for sensory stimuli

We expressed FlincG3 in sensory neurons that use cGMP as a second messenger and found that the sensor responds robustly to changing stimulus presentation. The increased fluorescence of FlincG3 at the sensory endings of AFD near a given T_c_ provides the first visual evidence that suggests cGMP dynamics in AFD are correlated with T_c_. These dynamics are consistent with the calcium traces of AFD during thermosensory transduction, with each peaking near T_c_ (Yu *et al.* 2014). In the gustatory neuron pair ASE, changes in FlincG3 fluorescence in response to NaCl concentration steps suggest that the sensor can respond reliably to acutely changing cGMP levels, providing, for the first time, visual evidence that cGMP levels in ASE are modulated by NaCl concentration changes. Importantly, we demonstrated that FlincG3 and jRGECO1a fluorescence changes in the ASER cell body require the rGC GCY-22. This suggests that GCY-22 is the primary rGC that produces cGMP for the NaCl response in ASER. Interestingly, the changes we observe in FlincG3 and jRGECO1a fluorescence are opposite in sign in the ASE cell body, which suggests that cGMP levels are inversely correlated with calcium in response to a NaCl concentration change. Previously reported genetic and calcium imaging evidence suggested that cGMP levels would directly correlate with calcium levels, as cGMP is hypothesized to bind to and open the cyclic nucleotide-gated cation channel TAX-2/TAX-4 to allow for calcium influx (Suzuki *et al.* 2008). A recent cryo-EM study also showed that cGMP gates the homomeric TAX-4 channel in the open state (Li *et al.* 2017). Additionally, physiological investigations of the heterologously expressed heteromeric TAX-2/TAX-4 channel indicated that it opens in response to cGMP binding (Komatsu *et al.* 1999; O’Halloran *et al.* 2017). There are a number of hypotheses for how a decrease in cGMP levels in the cell body may relate to the observed calcium increases in the same compartment of the cell. One hypothesis is that changes in cGMP levels in the sensory cilia are opposite in sign to changes in cGMP levels in the cell body. This has been previously reported in the olfactory neuron AWC, where cGMP in the sensory cilia decreases in response to the odorants isoamyl alcohol and benzaldehyde; in contrast, cGMP increases in the cell body in response to these odorants (Shidara *et al.* 2017). It is possible that this decrease of cGMP in the sensory cilia could lead to a decrease in calcium in AWC, consistent with its function as an OFF neuron (Shidara *et al.* 2017). Likewise, this may happen in ASE, where NaCl concentration upsteps (in the case of ASEL) and downsteps (in the case of ASER) may increase cGMP in the cilia and decrease cGMP in the cell body. In this case, cGMP in the cilia could bind to and activate TAX-2/TAX-4, which would lead to a calcium influx that could then be propagated throughout the cell through the opening of voltage-gated calcium channels. The potential transform in sign between the cilia and the cell body in ASE could be due to the activity of PDEs. For instance, the calcium-regulated PDE-1 has been shown to be required for a decrease in cGMP in the cell bodies of a subset of oxygen-sensing PQR neurons in response to a 7–21% increase in oxygen (Couto *et al.* 2013). Another hypothesis is that the primary sensory signal is carried not by the TAX-2/TAX-4 channel but by the PKG EGL-4, which has been shown to be required for sensing NaCl concentration changes in ASE (Suzuki *et al.* 2008). EGL-4 could be negatively regulating calcium channels; in this case, a decrease in cGMP would lead to the opening of these calcium channels. Testing these hypotheses, however, is beyond the scope of the present study.

There are at least three explanations for why *gcy-22* may be required for a reduction of cGMP in the ASER cell body in response to a NaCl downstep. First, the NaCl downstep may turn off GCY-22, which would result in a decrease in cGMP. In contrast, chronic loss of the GC would be recorded as a stable FlincG3 signal. Second, a NaCl downstep may increase GCY-22 activity, and, thus, cGMP, which can then bind to and activate a cGMP-regulated PDE such as PDE-2 (Couto *et al.* 2013). Once the PDE is activated, cGMP levels would decrease. In the absence of GCY-22, this decrease would not occur in response to a NaCl downstep, and a stable FlincG3 signal would be observed. Third, if GCY-22 activation increases cGMP levels, cGMP binding to the TAX-2/TAX-4 channel could lead to an influx of calcium. This could then activate a calcium-regulated PDE such as PDE-1, which would ultimately decrease cGMP. In contrast, the absence of GCY-22 would block changes in calcium in response to a NaCl downstep. Each possibility could be tested in a future study.

In the nociceptive phasmid neuron PHB, genetic evidence suggesting that it uses cGMP to signal the presence of SDS was corroborated by changes in PHB FlincG3 fluorescence. This is the first visual evidence for a cGMP-based signal in PHB, showing that it increases in response to an environmental cue.

Importantly, expression of FlincG3 did not perturb the function of AFD and PHB neurons, as negative thermotaxis and SDS repulsion was as robust in the FlincG3-expressing lines as in wild-type animals. This is in contrast to FlincG3 expression in ASE, which caused a slight preference for higher NaCl concentrations but minimally affected the plasticity of the NaCl concentration cultivation preference.

### Prospects for optimizing FlincG3 and extending its use

Like FlincG3 in ASE, the first generation of calcium sensors affected the behavior of neurons in which they were expressed: for instance, the FRET-based YC2.12 acted as a calcium sponge, a function that was exploited by Ferkey *et al.* (2007) to study nociceptive signaling, and the GFP-based GCaMP2.2 blocked olfactory plasticity (C. Brueggemann and N. L’Etoile, personal communication). Mutations that increased the quantal yield of GCaMP allowed the reporters to be expressed at lower levels that did not interfere with cellular function, yet were bright enough for imaging. Indeed, if FlincG3 was enhanced to mimic the properties of GCaMP6s, which contains (among other mutations) a K78H mutation in the cpEGFP domain that improved sensitivity relative to GCaMP3, the fluorescence might be bright enough to allow for lower expression of this reporter and thus reduce the possibility of it interfering with cellular functions (Chen *et al.* 2013). Until such optimizations are made, it will be necessary to select for lines that express FlincG3 at the lowest levels that allow for imaging, thereby minimizing the potential for behavioral effects. Addition of a subcellular localization signal may also mitigate off-target effects.

We think FlincG3 could be acting as a cGMP sponge due to its effects on NaCl-seeking behavior when expressed in ASE (Figure 6B and Figure S7). These behavioral results suggest that FlincG3 could be altering free cGMP levels in ASER, which could lead to tuning the NaCl concentration cultivation preference to be higher relative to nontransgenic siblings and wild-type animals. If FlincG3 can be shown to act as a cGMP sponge, this could also be exploited to specifically and locally perturb cGMP levels. For example, if one could localize a nonfluorescent form of FlincG3 at the cilia, this may perturb function in a different way from when it is localized to the cell body. This could reveal specific functions for cGMP signals at the sensory dendrites that are different from those in the cell body.

Additionally, the subcellular landscape of cGMP can also be probed using FlincG3. For instance, adding a small tag that localizes FlincG3 to specific regions of the cell along with a red protein for ratiometric imaging may reveal important aspects of the subcellular landscape of cGMP.

Though FRET-based cGMP sensors have been useful for uncovering cGMP dynamics in biological processes in an intact animal (Couto *et al.* 2013; Shidara *et al.* 2017), the single fluorophore FlincG3 provides the more accessible possibility of using additional fluorophores of different wavelengths to mark subcellular regions. This advantage of FlincG3 will provide a simple and powerful tool with which to visualize changes in cGMP concentration across the subcellular landscape. Additionally, the ability to simultaneously visualize cGMP and calcium by using FlincG3 with a red calcium sensor (Dana *et al.* 2016) allows us to investigate the dynamics of these second messengers at any marked subcellular location. Our results demonstrate that FlincG3 can be used to rapidly and specifically measure cGMP dynamics in the intact, behaving organism.
